# Novel agents and biomarkers for acute lymphoid leukemia

**DOI:** 10.1186/1756-8722-6-40

**Published:** 2013-06-18

**Authors:** Yanmin Zhao, He Huang, Guoqing Wei

**Affiliations:** 1 Bone Marrow Transplantation Center, The First Affiliated Hospital of Zhejiang University, No. 79 Qing Chun Rd, Hangzhou 310003, China

**Keywords:** Acute lymphoblastic leukemia, Genetic alteration, Philadelphia chromosome, Tyrosine kinase inhibitor, Monoclonal antibodies, Stem cell transplantation

## Abstract

New genetic markers for adult acute lymphoblastic leukemia (ALL) have been found to have prognostic impact, such as the lymphoid transcription factor gene IKZF1 alterations, which are associated with a high rate of leukemic relapse in B-ALL. Although complete remission rates by induction chemotherapy in ALL are now high, the long-term survival is still disappointing. Improvements in the survival outcome of ALL have been observed in young adults as a result of the use of pediatric inspired regimens and the broadening of the number of patients who are eligible for allogeneic SCT. Development of new and less toxic agents also provide promise to improve the outcome in adult ALL, such as tyrosine kinase inhibitors in Ph-positive ALL, rituximab in CD20-positive disease, blinatumomab in precursor B-ALL and nelarabine in T-lineage ALL. Challenges for the future are to implement genomic profiling into the clinical setting to guide risk stratification and providing novel targets for tailored therapies.

## Introduction

Acute lymphoblastic leukemia (ALL) is a biologically and clinically heterogeneous neoplasm of lymphoid progenitors, with approximately 85% of cases being of B-cell lineage and 15% of T-cell lineage. In contrast to childhood ALL, in whom approximately 90% are now cured [1], adults with ALL usually carry a worse prognosis with a long-term survival rate less than 35-40% [2], even with allogeneic hematopoietic stem cell transplantation (allo-SCT). The current understanding of the biologic determinants of treatment failure in ALL is limited, and the treatment of refractory or relapsed ALL remains a major challenge. By summarizing recent dvelopments and particularly the highlights from the 2012 ASH Annual Meeting, this study will review some latest advances in the biological features of adult ALL, with an emphasis on the role of genetic alteration on prognosis of this malignancy and treatment approaches to both Philadelphia chromosome-positive (Ph+) and negative (Ph-) ALL.

### Integration of genetic markers into risk stratification algorithms

The inherent heterogeneity of ALL requires an accurate assessment of risk to aid treatment decisions. In the past, the classic prognostic factors were age, presenting white blood cell (WBC) counts, cytogenetic abnormalities and upfront response to induction therapy. One of the strongest adverse prognostic features is the presence of the Ph chromosome t(9;22). Recently, a retrospective analysis of the Mayo Clinic leukemia database has identified high risk cytogenetics [−7, del(7p), +8, MLL translocations, t(1;19), t(17;19), t(5;14)], very high risk cytogenetics categories [t(4;11), t(8;14), complex (≥ 5 abnormalities), hypodiploidy, triploidy] [3], low platelet counts and poor Performance Status (PS) at diagnosis were independent predictors of inferior outcomes in patients with Ph-negative ALL [4]. Detection of minimal residual disease (MRD) is also used to identify high-risk patients [5]. These prognostic clinical features of ALL were summarized here in Table 1.

**Table 1:** Prognostic clinical features of ALL

**Risk factors**	**Prognosis association**
At presentation	
Age	Adverse outcome with advancing age
CNS involvement	Adverse outcome
Presenting WBC count	Adverse for B-ALL > 30
	Adverse for T-ALL >100
ECOG PS	Poor PS at diagnosis were an independent predictor of inferior outcomes
Cytogenetics	Favorable: hyperdiploidy
	Adverse: t(9;22), t(4;11), t(8;14), complex (≥ 5 abnormalities), hypodiploidy, triploidy, -7, del(7p), +8, MLL translocations, t(1;19), t(17;19), t(5;14)]
In response to therapy	
Time to initial response	Adverse: failure to attain complete remission within 4 weeks of induction
Detection of MRD	Adverse: detection at various time-specific points in several studies

Nowadays, microarrays and next generation sequencing provide new approaches to profile ALL genomes. These studies have identified some new subtypes of ALL harboring recurring submicroscopic genetic alterations, several of which have clear implications for risk stratification and targeted therapeutic intervention.

Till now, more than 50 recurring genetic alterations have been identified, and many of the genes involved encode proteins with key roles in lymphoid development (PAX5, IKZF1, and EBF1), transcriptional regulation (ETV6, ERG), lymphoid signaling (BTLA, CD200, TOX, BLNK, VPREB1), cell-cycle regulation and tumor suppression (CDKN2A/CDKN2B, RB1, PTEN), and drug responsiveness (the glucocorticoid receptor NR3C1) [6]. These specific genetic alterations cooperate in leukemogenesis, however, few have been found to have definite prognostic impact, with the notable exception of alterations of the lymphoid transcription factor gene IKZF1 in B-ALL.

IKZF1 encodes IKAROS, a zinc finger transcription factor that is required for the development of all lymphoid lineages [7]. During the last 5 years, IKZF1 has been identified as one of the most clinically relevant tumor suppressors in ALL [8,9]. Deletion or sequence mutation of IKZF1 are detected in more than 70% of BCR-ABL1+ lymphoid leukemia, including de novo ALL and chronic myeloid leukemia at progression to lymphoid blast crisis, [10] and are associated with poor outcome in BCR-ABL1+ adult ALL [11]. Moreover, a subset of BCR-ABL1 negative B-ALL patients with IKZF1 alterations exhibit a gene-expression profile similar to BCR-ABL1+ ALL, named as BCR-ABL1–like ALL. These cases may have alternative genetic events resulting in aberrant activation of tyrosine kinase signaling pathways similar to those downstream of BCR-ABL1, including rearrangement of CRLF2 and activating mutations of Janus kinases (JAK1/2) [12,13]. The prognosis of BCR-ABL1–like ALL is poor. In the COG AALL0232 study of high-risk B-progenitor ALL, the event-free survival (EFS) for BCR-ABL1–like cases was 62.6% ± 6.9% compared with 85.8% ± 2.0% for non–BCR-ABL1–like cases (P < 0.0001) and was associated with poor outcome after adjustment for age, sex, WBC at presentation, and levels of MRD [14].

In addition to IKZF1, other genetic alterations were also identified to have some prognostic impact on ALL and incorporated into risk stratification algorithms (Table 2). For example, B-ALL cases with CRLF2 rearrangements, JAK1/2 mutations [12], CDKN2A/B deletions [15], TP53 deletions/mutations [16], or CREBBP deletions/mutations [17] had inferior outcomes in terms of response to chemotherapy, overall survival, or incidence of relapse. For adult T-ALL, NOTCH1 and/or FBXW7 (N/F) mutations were associated with a favorable outcome [18,19], while N/K-RAS mutations or PTEN deletions/mutations demonstrated trends to a worse outcome [20,21]. Therefore, Trinquand A et al. propose a new T-ALL oncogenetic classifier defining low-risk patients as those with N/F mutation but no RAS/PTEN mutation, allowing identification of nearly 50% very good prognosis T-ALL adults [21]. Recently, mutations in the cytosolic 5′-nucleotidase II gene (NT5C2), were identified to drive resistance to treatment with nucleoside analog therapies in T cell or B-precursor ALL [22].

**Table 2:** Novel key genomic alteration associated with prognosis in adult ALL

**Gene alteration**	**Frequency**	**Comments**
IKZF1 deletions and sequence mutations	15% of pediatric B-ALL cases; 70% of BCR-ABL1 + lymphoid leukemia, and 30% of high-risk BCR-ABL1-like B-ALL	IKZF1 alterations are associated with poor outcome in both BCR-ABL1–positive and negative ALL cases, and triple the risk of treatment failure. IKZF1 status is an independent risk factor at a multivariable analysis of established prognostic factors.
CRLF2 rearrangement (as IGH@-CRLF2 or P2RY8-CRLF2)	Up to 16% of pediatric and adult B-ALL; >50% Down syndrome (DS) ALL	Concomitant JAK1/2 mutations in >50% of cases; associated with IKZF1 alteration and poor outcome, particularly in non-DS-ALL.
JAK1/2 mutations	Up to 10% of high-risk BCR ABL1-like B-ALL; 18–35% of DS ALL	Almost all cases of B-ALL with JAK1/2 mutations harbor concomitant CRLF2 rearrangement, associated with poor outcome; may be responsive to JAK inhibitors.
CREBBP deletions and sequence mutations	19% of relapsed B-ALL	Associated with glucocorticoid resistance; Resulted in impaired acetylation of histone targets; histone deacetylase inhibitors may be useful.
CDKN2A/B deletions	~30% of B-ALL; 47% of relapsed BCR-ABL1-ALL;	Associated with poor outcome in terms of overall survival, and incidence of relapse in adult BCR-ABL1-positive ALL; controversial prognosis in other B-ALL subtypes.
TP53 deletions and sequence mutations	Up to 12% of B-ALL;	Enriched at relapse and associated with non-response to chemotherapy and poor event-free survival and overall survival.
PHF6 deletions and sequence mutations	38% of adult T-ALL cases	Associated with reduced overall survival.
PTEN deletions and sequence mutations	6–8% of T-ALL	Associated with poor response to chemotherapy and resistance to pharmacological inhibition of NOTCH1
N/K-RASmutations	10% of adult T-ALL	N/K-RASmutations demonstrated trends to a worse outcome.
NOTCH1 mutations	~50% of T-ALL	Associated with favorable outcome
FBXW7 mutations	12-24% of adult T-ALL	Associated with favorable prognosis due to enhanced glucocorticoid receptor α levels and steroid sensitivity
NT5C2 mutations	19% of relapse T cell ALL and 3% of relapse B-precursor ALL	NT5C2 mutant proteins increase nucleotidase activity in vitro and drive resistance to treatment with nucleoside analog therapies

### Current treatment options for treatment of Ph-positive acute lymphoblastic leukemia

Philadelphia chromosome/BCR-ABL–positive ALL has long been recognized as a high-risk subset of adult ALL with the most unfavorable prognosis. Published data show a disappointing long-term survival of 20% or less with chemotherapy alone [23]. Introduction of the tyrosine kinase inhibitors (TKIs), most commonly imatinib (IM) in combination chemotherapy has led to an encouraging treatment outcome, [24-28] with complete remission rates exceeding 90% and estimated overall survival (OS) ranging from 30% to 70%. The proportion of patients able to proceed to and benefit from allo-SCT in CR1 has increased with TKI-based therapy, and there is no evidence that IM has an adverse effect on transplant-related morbidity or mortality. Nevertheless, there are still many challenges related to this disease, including the selection of appropriate pre-transplant therapy, the correct use of TKIs after transplant, and development of potential therapeutic strategies to overcome disease resistance. Furthermore, the incidence of Ph + ALL increases with age, and a significant proportion of elderly patients are not eligible for SCT, in whom alternative approaches should be explored.

### Therapeutic strategies for ‘transplantable age’ patients

It is well established that TKI-based treatment, followed by allo-SCT in CR1, is the gold standard therapy and provides curative potential for adults with Ph + ALL. Studies from several cooperative groups, have showed 3-year survival probabilities of 55–70% in young adults receiving IM-containing induction followed by myeloablative allo-SCT [27,28]. And the survival is inferior in non-transplanted patients, despite the inclusion of IM in the protocols. More recently, the Northern Italy Leukemia Group disclosed a long term results of patients who were treated with chemotherapy plus short IM pulses. In the study, the probability to receive an SCT was significantly increased in IM + patients compared with IM- cohort (63% vs. 39%, p = 0.04), indicating that incorporation of IM to chemotherapy not only increase the CR rate, but also increase the number of patients proceeding to SCT. And patients receiving a SCT had the best outcome (n = 58; 5-years OS 49%), while in those unable to reach SCT, outcome was inferior (IM + SCT-: n = 17, 5-years OS 11%; IM-SCT-: n = 13, 5-years OS 8%), indicating allo-SCT remain the best consolidation option even in patients who achieve a good response to TKI-based therapy [29].

Of note, there is increasing reluctance to offer allo-SCT to younger patients in the TKI era, because younger individuals usually have a better outcome to chemotherapy/TKI alone and have more to lose by risking the long-term adverse consequences of SCT. In a previous study, Schulz et al. found that intensive chemotherapy plus continuous IM improved survival rate for children and adolescents, with minimal toxicities [30]. And the 3-year EFS was not significantly different for the patients receiving chemotherapy/IM alone compared to those proceeding to sibling donor SCT (88% ± 11% vs. 57% ± 22%). This study suggested the outcomes were not superior with allo-SCT compared with intensive chemotherapy plus IM for a subgroup of younger patients. Recently, Santhosh et al. also evaluated the feasibility of combining IM with a pediatric-based regimen (a modified version of the Dana Farber Cancer Institute protocol) for young adults with Ph + ALL, in whom the complete response rate was 94%. Of 16 patients who underwent allo-SCT in CR1, six died of non-relapse complications. Based on an intention-to-treat and time-to-donor identification analysis, they did not find any significant difference in either OS or EFS between transplanted and non-transplanted patients [31].

Nevertheless, interpretation of these findings is limited by the small patient numbers and the relative short follow-up. Thus, it is difficult to answer whether IM/intensive chemotherapy could replace allo-HSCT for younger patients with Ph + ALL. However, these provocative data open the way for further discussion. Future studies will focus on development of independent prognostic algorism, such as MRD at relevant time-points, to identify a subgroup of good risk younger patients, eligible for receiving treatment with chemotherapy and TKI only, and accordingly sparing SCT.

### Imatinib-based therapy in elderly patients

Ph + ALL accounts for 40 – 50% of ALL in patients over 50 years, many of whom cannot tolerate intensive chemotherapy and are not eligible for SCT. The question of whether minimization of therapy-related toxicity by combining TKIs with less intensive chemotherapy yielded equivalent results were proposed in this subgroup of patients. Several studies showed IM combined with minimal chemotherapy was able to yield a higher CR rate and fewer deaths without major toxicity in elderly patients than observed in historical controls. However, a main problem of this less intensive approach is the very high relapse rate, yielding an OS probability of less than 20% in the studies with long-term follow-up [32]. Dasatinib is a dual BCR-ABL and Src kinase inhibitor with approximately 325-fold greater activity than IM against BCR-ABL, and with activity against most mutations resistant to IM, with the exception of T315I, but to date, it is still unknown whether dasatinib therapy is superior to IM in terms of preventing relapse or inducing longer EFS. Now, the European Working Group on Adult ALL (EWALL) disclosed the final results of the combination of dasatinib and a low Intensity chemotherapy (vincristine and dexamethasone) for first-Line treatment in patients with de novo Ph + ALL aged > 55 years [33]. This regimen was followed by chemotherapy and dasatinib interspersed between the therapeutic cycles. Of the 71 subjects, 94% achieved a CR. At 3 years, relapse free (RFS) and overall survival (OS) were estimated as 42.7% and 44.7%, respectively. 29 patients relapsed after a median of 9 months. Most relapses were associated with the T315I mutation. And the presence of abnormalities additional to t(9;22) were associated with a worse RFS. To some extent, the 3-year OS at 44.7% for elderly patients is quite encouraging, a result in the range of reported survival in younger adults. This study also identified a subgroup of elderly patients with poor prognosis, in whom reduced intensity conditioning (RIC) or non-myeloablative HSCT will be considered to perform as the consolidation therapy. Other studies incorporated nilotinib or interferon to the induction or consolidation therapy and tried to determine whether these new regimen would improve the results for the elderly, without increasing the toxicity. An Italian trial enrolled 39 Ph + ALL patients aged > 60 years or unfit for intensive chemotherapy and SCT [34]. They were treated with two TKIs (nilotinib 400 mg twice daily, and imatinib 300 mg twice daily), alternating for 6 weeks for a minimum of 24 weeks. Overall, one patient was primarily resistant and 13 patients relapsed, and the OS at is 64% at 2 years. Although in this small cohort of elderly/unfit patients, the rates of relapse and progression were not likely to be different from the rates observed with imatinib alone [35] and dasatinib alone [36], It’s important to notice that the mutations that occurred at the time of relapse were sensitive to other TKIs (dasatinib and ponatinib).

Interferon as treatment for Ph + leukemia has attracted renewed interest, fueled by preclinical observations that IFN may recruit dormant CML stem cells into the cell cycle, and enhance their susceptibility to eradication by TKIs. More available clinical data come from the treatment of CML. Recently, an open-label phase II study was designed to investigate the combination of low-dose IFN-alfa with IM as maintenance therapy (MT) in the elderly with Ph + ALL patients [37]. Maintenance therapy consisted of IM at a single dose of 600 mg daily, combined with low-dose subcutaneous interferon-alfa-2a starting at 1 MU three times a week with dose escalation to a target dose of 3 MU three times a week. Median overall survival for patients (n = 7) receiving IM + IFN-alfa is 5.4 vs. 2.9 years for patients receiving IM alone (n = 12). The patients receiving IM + IFN- alfa had a longer median remission duration from start of maintenance (2.2 years), compared with patients receiving IM as MT (0.75 yrs, p = 0.07). The lack of relationship between number of consolidation cycles and remission duration suggests IM + IFN-a MT may be effective even if started earlier during front-line therapy. Evaluation of the more potent 2nd generation TKI in combination with IFN-alfa for Ph + ALL is warranted in future.

### Administration of TKI after SCT

Minimal residual disease after allo-SCT for Ph + ALL is predictive of relapse. Imatinib administration subsequent to SCT may prevent relapse, but to date there is no large-scale prospective randomized control trial to provide sufficient evidence to conclude that IM or other TKIs should be given to all patients after SCT, even though small studies suggested efficacy [38,39]. A single center study from the University of Minnesota showed a trend toward improved outcome in patients who could be treated with IM in the pre- and post-transplant period following myeloablative conditioning [40]. Ram et al. also reported that IM given for a median duration of 1 year after RIC-SCT had good tolerability and was associated with an improved outcome, although the effect on relapse was not statistically significant [41]. In a Chinese study, IM treatment was scheduled for 3–12 months after SCT, until BCR-ABL transcript levels were negative at least for 3 consecutive tests or molecular remission was sustained for at least 3 months. It was proven that administration of IM after SCT could reduce the 5-year relapse rate (IM group vs. non-IM group: 10.2% vs. 33.1%, p = 0.016), and prolong the 5-year DFS. (IM group vs. non-IM group: 81.5% vs. 33.5%, p = 0.000) [38]. Similarly in a Japanese study, IM after allo-SCT also had a favorable impact on outcome [42].

The optimal time for initiating IM treatment following SCT is not well established. The CSTIBES02 trial reported that IM was poorly tolerated following myeloablative SCT: only 62% were able to start at a median of 3.9 months after SCT, and many patients required discontinuation or dose reduction due to complications such as GVHD [24]. The GMALL study compared the tolerability and efficacy of post-transplant IM administered either prophylactically (to begin at 3 months after SCT) or following any BCR-ABL reappearance. No significant difference in outcome was observed between prophylactical IM or MRD-triggered IM after SCT (5-year OS 82% vs. 78%, respectively). However, more than half of enrolled patients discontinued IM in both groups, predominantly owing to gastrointestinal toxicities, indicating poor tolerance of IM when given early after allo-SCT [43]. In a recent study, post-SCT IM therapy was initiated if patient neutrophil counts were >1.0 × 10^9^/L and platelet counts were >50.0 × 10^9^/L, or if they displayed either elevated BCR-ABL transcript levels in two consecutive tests, or a BCR-ABL transcript level ≥ 10^-2^ after initial engraftment [38]. In this design, interruptions of IM therapy due to adverse events (AEs) were relatively lower. Thus, careful consideration should be given as to when to start IM, based on patient BCR-ABL transcript levels, while concurrently taking into account the clinical conditions of individual patients (including engraftment and GVHD).

Experience on the use of dasatinib after HSCT is very limited. A study by Caocci and colleagues suggested that administration of dasatinib 120 days after SCT is highly effective in preventing Ph + ALL relapse after both auto- and allo-SCT [44]. However, another preliminary data from Taiwan could not fully support this conclusion, and extramedullary relapse could be problematic despite provision of both allo-SCT and dasatinib [45]. Prospective and randomized studies for indentifying the appropriate TKI scheme in Ph + ALL after SCT are urgently needed.

### Future treatment to overcome TKI resistance

Despite high remission rate induced by TKI-containing regimens, fewer than 50% of these patients sustain molecular remission after initial therapy, and overt clinical relapses are frequent, indicating that TKI resistance is a substantial obstacle in Ph + ALL. The emergence of BCR–ABL mutations is the most important mechanism of resistance to TKI. Mutations can occur within the A (activation)-loop, the P (ATP-binding)-loop region or at the so-called “gatekeeper” residue, threonine 315. Given that the prognosis of patients with Ph + ALL with T315I mutation is poor, many new agents have been clinically investigated in the setting of T315I and other mutations. Ponatinib is a pan-BCR-ABL inhibitor with activity against all IM-resistant mutants, including the T315I mutation. Ninety four patients with CML lymphoid blast phase or Ph + ALL were included in the pivotal phase 2 study, who were resistant or intolerant to other TKIs [46]. With a short median follow up of 2 months, the major hematologic response were 37% and 27%, in patients with resistance/intolerance to prior TKI and those with T315I mutation, respectively. Treatment with ponatinib was generally well tolerated, and thrombocytopenia and neutropenia were the most common grade 3 or more AEs. Thus, ponatinib combined with chemotherapy is a promising approach to overcome TKI resistance. Other new drugs such as Aurora kinases (AK) inhibitors [47]and DCC-2036 [48] have recently entered preclinical or clinical testing, but more mature data are required.

### New treatment approaches for Ph -negative acute lymphoblastic leukemia

Although cure rates in pediatric ALL are now around 90%, there is still room for improvement in the management of adult patients with Ph- ALL, where little progress has been made over the past two decades and long-term cure rates usually do not exceed 40%, even with allo-SCT [49]. Given the disappointing outcomes, there is a critical need for developing new approaches to treating adults with this disease, including the use of “pediatric inspired” regimens and incorporation of novel targeted agents alone or in combination with other chemotherapeutic drugs.

### Application of pediatric inspired regimens to young adults

One of promising approaches in treating young patients with Ph- ALL is adopting pediatric protocols - the so called “pediatric inspired” regimens, especially for those adolescent and young adult (AYA) aged 15 to 39 years. Retrospective studies have shown that AYA with ALL treated with pediatric-inspired regimens have better outcomes than similarly aged patients with adult protocols, and improved the event-free survival rate of newly diagnosed Ph- ALL to 60% or higher [50]. This compared to 35%–40% for historical controls and without additional benefit of SCT. However, prospective studies comparing AYA adopting adult protocols with pediatric protocols are rare. Recently, a Japan adult leukemia study group conducted a phase II multicenter study to determine the outcome of pediatric protocols for AYA with Ph- ALL (Japan Adult Leukemia Study Group (JALSG) ALL202-U study) [51]. In comparison with the former JALSG ALL-97 adult protocol, the pediatric protocols showed an increase in cumulative dose of cyclophosphamide (1.5-fold), vincristine (1.2-fold), L-asparaginase (18-fold) and methotrexate (3.7-fold), respectively. As for the intrathecal chemotherapy, the duration was longer and the frequency was increased from 8 to 15 times. Of the 138 eligible patients, 134 patients (97.1%) achieved CR. Compared with previous JALSG ALL-97 study, CR rate (97.1% ALL202, 84% ALL97; p = 0.01), 4-year DFS rate (71% ALL202, 46% ALL97; p = 0.0001) and 4-year OS rate (74% ALL202, 46% ALL97; p = 0.0002) were significantly higher in this pediatric protocols. Studies by groups in other countries also yielded comparable results [52]. Although it is still unknown why treatment with pediatric protocols resulted in improved outcomes, the disparity may be explained by some common features of pediatric treatment strategies, including (1) significantly increasing the non-myelosuppressive agents such as vincristine and steroids, (2) using much higher cumulative doses of asparaginase (ASP) for prolonged asparagine depletion, (3) administering very early and more frequent intrathecal methotrexate together with very high-dose systemic methotrexate, (4) including longer antimetabolite-based maintenance cycles, and finally, (5) patients treated on pediatric protocols are more likely to adhere to cycle start dates and have shorter rest between cycles. Together, these features may contribute to the improved outcomes for young patients treated on pediatric protocols. However, the outcomes for AYAs with ph- ALL still lag significantly behind those achieved in younger children. Thus, challenges for the future include identifying unique aspects of drug pharmacology in young adults to refine dosing schedules, and recognizing additional biologic factors of AYA, with the anticipation that these discoveries will provide new targeted therapeutic opportunities.

### Novel and emerging drugs for ph-negative ALL

Classic treatment regimens using combination chemotherapy are associated with substantial toxicities that limit further dose escalation. Therefore, development of new, targeted and less toxic agents, such as nucleoside analogues and monoclonal antibodies (mAbs), offer promise for improving the outcome of Ph- ALL.

### New types of asparaginase

As mentioned above, young adults with ALL adopted pediatric regimens, with prolonged use of ASP, mostly E. coli, had better outcomes than historically reported. Considering its potential higher toxicity, however, ASP is either not used or given for much shorter duration, particularly in patients aged > 45 years. Some new types of ASP are emerging. The pegylated E. coli derived form, PEG-ASP, is most commonly used, because it shows less immune response and a prolonged half-life of 5.7 days compared to 1.3 days for native ASP. The incorporation of PEG-ASP in induction and especially during consolidation therapy seems to improve the outcome with a manageable and reversible toxicity [53,54] as shown in several studies. However, clinical hypersensitivity reactions still occur in 10-30% of patients requiring its discontinuation. Asparaginase Erwinia Chrysanthemi (Erwinaze™) is an L-ASP derived from a different bacterium and is immunologically distinct from the E. coli L-ASP. Plourde et al. conducted a compassionate use trial of Erwinaze in 1368 ALL patients with hypersensitivity to native E. coli or PEG-ASP to collect safety information [55]. 77.6% of patients were able to complete their Erwinaze treatment. Discontinuation due to allergic reaction occurred in 8.8% and other AEs in 4.7%. The study suggested Erwinaze was well tolerated with no unexpected toxicities identified beyond those associated with L-ASP treatment. In addition to Erwinaze, L-asparaginase-loaded red blood cells (GRASPA®) has been a new available option for ALL population, including older patients with the disease. In a randomized trial from France, the use of GRASPA in refractory or relapsing ALL showed a reduction in the number and severity of allergic reactions and a trend to fewer coagulation disorders [56]. Another phase II dose escalation study aimed at determining optimal dose of GRASPA that could be combined with standard EWALL chemotherapy backbone in patients aged >55y with newly diagnosed Ph- ALL [57]. The study indicated that GRASPA at a dose of 100 UI/kg infused twice during induction cycles, appears to be the best manner with sustained asparagine depletion and a good efficacy/safety profile, and the dose of 100 IU/kg was associated with median OS of 15.6 months, compared favorably with historical control.

### Monoclonal antibodies

Recently, the development of monoclonal antibodies has provided hope that tumor-targeted therapy would one day play a role in the treatment of ALL with less systemic toxicity profile [58]. To date, the most data are available for anti-CD20 (rituximab), which has been combined with chemotherapy for the treatment of B-lineage ALL or Burkitt lymphoma [59]. In an update of study from M. D. Anderson, 216 pts with Ph- B-lymphoblastic leukemia have been treated with the modified hyper-CVAD regimens with or without rituximab [60]. For the younger (age < 60 years) CD20-positive subset, rates of complete remission duration (CRD) and OS were better with the combination of hyper-CVAD plus rituximab than with hyper-CVAD alone (69% v 38%; P < .001% and 71% v 47%, P = .003). However, in contrast to the Burkitt experience, no benefit from the addition of rituximab was noted in elderly patients (age > = 60 years), in part because of deaths in CR from infections during consolidation. A similar outcome was also observed in CD20+ Ph- B-precursor ALL from the German GMALL 7/03 study [61]. In their study, among patients younger than 55 years, the addition of rituximab was associated with a 5-year survival rate of 71% versus 51% without rituximab. These studies suggest that rituximab added to intensive chemotherapy has improved the outcome for CD20-positive B-lineage ALL, particularly among younger adults. Further investigation will address the role of extended rituximab therapy with attenuated chemotherapy (to reduce risk of infectious complications) in older patients. And studies combining chemotherapy with ofatumumab or with other more potent CD20 monoclonal antibodies such as GA-101 are also warranted [59,62,63].

Another actively investigated MoAb is anti-CD22. Epratuzumab, the “naked” humanized MoAb against CD22, was first studied in children with relapsed ALL showing minimal single-agent activity [64]. For adult patients, a phase II study through the Southwest Oncology Group (SWOG) is ongoing and presenting some findings. They found giving epratuzumab together with cytarabine and clofarabine improving response rate (to 45%) in patients with relapsed/refractory (R/R) precursor B-ALL, and thus warrants further testing [65]. Inotuzumab ozogamicin, a CD22 monoclonal antibody bound to calicheamicin, is also active in ALL. In a phase II study of inotuzumab 1.3 to 1.8 mg/m^2^ IV once every 3 to 4 weeks in 49 patients with R/R B-lineage ALL, the overall response rate was 57% and the median survival of all patients was 4.5 months. Given the heavily pretreated patients, the response rate in R/R ALL was promising and twenty-two of the 49 patients were able to proceed to subsequent allo-SCT. The most common adverse effect was liver function abnormalities that were severe in 31% of the patients [66]. Recently inotuzumab was delivered at smaller doses and a more frequent schedule (inotuzumab weekly, 0.8 mg/m2 D1, 0.5 mg/m2 D8 and 15, q3-4 weeks) than previously studied. The modified schedule appears to be equally effective with response rate of 53% and median survival of 6.3 months, and it is less toxic than single dose, indicating this weekly schedule might be superior to single dose schedule [67].

Bi-specific MoAbs constitute a new generation of compounds with a promising future. In a recent phase II trial, blinatumomab, a bi-specific MoAb with a CD3-binding site for the T cells and a CD19 site for the target B cells [68], has been highly effective in treating adult B-precursor ALL in hematological CR but with molecularly persistent or relapsed disease (MRD positive) [69]. More recently, blinatumomab was investigated in adults with active refractory/relapsed ALL. In an ongoing study, 36 adult patients received blinatumomab at three dosing regimens. Twenty six patients (72%) achieved a hematological CR or CR with partial hematological recovery, 24 of whom were MRD negative within the first 2 cycles. These results revealed blinatumomab may become the most effective new single targeted agent so far developed for B-lineage ALL. And side effects, including cytokine release syndrome and CNS events were manageable and reversible. As final dose and schedule, 5 μg/m^2^/day in week 1 and 15 μg/m^2^/day for week 2–4 was selected for further investigation based on safety/efficacy considerations [70].

### Other potential new agents

In addition to mAbs, other potential new agents are mammalian target of rapamycin (mTOR) inhibitors, JAK inhibitors [71] and the purine analogs. In T-ALL, attention must be paid to the novel purine analogs such as clofarabine, forodesine, and, especially, to nelarabine [72]. The latter drug has proven to be active in relapsed/refractory disease [73], and is being evaluated in combination with hyper-CVAD for newly diagnosed T-ALL patients [74].

### Improvement in SCT for ph-negative ALL

Allogeneic SCT is the most potent postremission anti-leukemic therapy in ph- adult ALL, with OS rates exceeding 50%. Several older randomized trials and meta-analyses concluded that high-risk, not standard-risk, patients benefited from allogeneic SCT in CR1 [75]. Modern protocols tend to avoid SCT in standard-risk patients who are confirmed to be MRD-negative given the excellent results of pediatric-based chemotherapy. However, two recent randomized trials from MRC-ECOG and JALSG give an opposite conclusion that the statistically significant survival advantage with allo-SCT appears to be greater for patients with standard-risk rather than high-risk ALL patients, a finding that likely reflects the increased treatment-related mortality (TRM) in the high-risk (especially older) patients that negated the GVL effect in these patients [49,76]. It is noted that inclusion of the younger patients in the two randomized trials may have biased the results in favor of allo-SCT, and therefore ongoing reassessment of the place of allo-SCT needs to be continued.

The increasing use of unrelated donors, umbilical cord blood (UCB), haploidentical donors, and reduced intensity SCT have expanded the opportunity for more patients to undergo SCT. The OS rate of unrelated donor (URD)-SCT are currently close to those for related donors; the higher transplant-related mortality of the former is counterbalanced by the lower relapse rate [77]. Development of high-resolution HLA typing technology will further improve the transplant outcome for URD-SCT. UCB has emerged as a major, untapped source for SCT, providing clinicians and patients with increased choices for alternative donors. The use of less well-matched UCB grafts has yielded equivalent results as 8/8 and 7/8 URD allele-matched grafts, enhancing the appeal of this product [78]. Although haploidentical SCT remains investigational, this approach has shown promise when used in conjunction with high-dose posttransplantation cyclophosphamide in patients with very high-risk ALL who lack an unrelated donor [79,80]. Finally, RIC facilitates SCT in elderly patients and those who have comorbid conditions. It is shown that RIC-SCT is associated with comparable donor engraftment rates and lower incidences of TRM, but with higher relapse rates than myeloablation. Thus, OS rates are similar to myeloablative conditioning. Given the negative inherent patient selection criteria bias, RIC-SCT could actually prove to be superior. Autologous SCT has a low antileukemic effect in adult ALL, but could be active in certain situations such as MRD negativity after consolidation [81].

## Conclusions

Despite outstanding advances in the knowledge of the molecular basis of adult ALL, the treatment of this disease remains challenging. Improvements in the outcome of ALL have been observed in young adults as a result of the use of inspired regimens and the broadening of the number of patients who are eligible for allogeneic SCT, whereas the prognosis of the elderly remains poor. Development of new and less toxic agents provide promise to improve the outcome in adult ALL, such as tyrosine kinase inhibitors in Ph-positive ALL, rituximab in CD20-positive disease, blinatumomab in precursor B-ALL and nelarabine in T-lineage ALL. From the biological prospective, genetic profiling approaches will provide a comprehensive view of the complexity of genetic alterations in ALL. Identifying miRNAs, genes, and pathways relevant for the pathogenesis of the leukemia and the biologic determinants of treatment failure may provide novel insights on novel targets [82]. However, these findings should be brought from bench to bedside for novel targeted therapies.

## Competing interests

The authors declare that they have no competing interests.

## Authors’ contributions

GW participated in concept design and critically revising the manuscript. YZ participated in data collection and drafting the manuscript. HH participated in reviewing and revising the manuscript. All authors have read and approved the final manuscript.
